# Microbiome-host interactions in locally advanced cervical cancer: the impact on chemoradiotherapy treatment outcomes and toxicity

**DOI:** 10.3389/fimmu.2026.1876914

**Published:** 2026-08-25

**Authors:** Helena C. J. Schellekens, Bob J. R. Bindels, Irene Regueiro Zapico, Servaas A. Morré, Evert J. van Limbergen, Edith M. G. van Esch, John Penders, Peggy J. de Vos van Steenwijk

**Affiliations:** 1 Department of Obstetrics and Gynecology, Maastricht University Medical Centre (MUMC+), Maastricht, Netherlands; 2 GROW – Research Institute for Oncology and Reproduction, Maastricht University, Maastricht, Netherlands; 3 Department of Obstetrics and Gynecology, VieCuri Medical Centre, Venlo, Netherlands; 4 Department of Genetics and Cell Biology, Faculty of Health, Medicine & Life Sciences (FHML), Maastricht University, Maastricht, Netherlands; 5 Dutch Chlamydia trachomatis Reference Laboratory, Department of Medical Microbiology, Infectious Diseases and Infection Prevention, Care and Public Health Research Institute (CAPHRI), Maastricht University Medical Centre (MUMC+), Maastricht, Netherlands; 6 Department of Molecular and Cellular Engineering, Jacob Institute of Biotechnology and Bioengineering, Sam Higginbottom University of Agriculture, Technology and Sciences, Allahabad, India; 7 Department of Radiation Oncology (MAASTRO), GROW Research Institute for Oncology and Reproduction, Maastricht University Medical Centre+ (MUMC+), Maastricht, Netherlands; 8 Department of Obstetrics and Gynecology, Catharina Hospital Eindhoven, Eindhoven, Netherlands; 9 Department of Obstetrics and Gynecology, Erasmus MC, Rotterdam, Netherlands; 10 Department of Medical Microbiology, Infectious Diseases and Infection Prevention, Maastricht University Medical Centre (MUMC+), Maastricht, Netherlands; 11 NUTRIM – Institute of Translational Research in Metabolism, Maastricht University, Maastricht, Netherlands

**Keywords:** chemoradiotherapy, immune microenvironment, locally advanced cervical cancer, microbiome, microbiome-targeted interventions, toxicity

## Abstract

Cervical cancer remains one of the most common malignancies among women worldwide, and treatment of locally advanced disease is often associated with substantial toxicity that negatively affects the quality of life. Therefore, strategies aimed at improving survival while minimizing treatment-related side effects are essential. Emerging evidence suggests that the gut and vaginal microbiomes influence cervical carcinogenesis, treatment response, and treatment-related adverse effects. This narrative review aims to summarize current evidence regarding the role of the gut and vaginal microbiomes in locally advanced cervical cancer and explores their potential clinical implications, including interactions with immunological mechanisms. Dysbiosis has been associated with chronic inflammation, impaired antitumor immune responses, reduced treatment efficacy, and increased toxicity, whereas a beneficial microbial composition appears to support improved therapeutic outcomes and reduced toxicity. In addition, microbiome-targeted interventions, including probiotics, show promise in modulating microbial composition, optimizing treatment outcomes, and mitigating treatment-related toxicity. Longitudinal studies integrating analyses of both the gut and vaginal microbiomes with immune infiltrates, patient-reported outcomes, and clinical treatment results are essential to clarify the therapeutic potential of microbiome-targeted interventions in personalized cervical cancer care. Particular attention should be given to interactions between the microbiome and immunotherapy, as immune checkpoint inhibitors are increasingly being incorporated into treatment strategies for locally advanced disease.

## 1 Introduction

Despite improvements in screening, cervical cancer remains one of the most common cancers among women worldwide, with approximately 660,000 new cases and 350,000 deaths reported in 2022 (1). Infection with high-risk Human Papillomavirus (hrHPV) occurs in up to 80% of sexually active women throughout their lifetime, and persistent hrHPV infection is the primary etiologic factor of cervical cancer (2). Standard treatment is stage-dependent: early-stage disease is usually managed surgically, whereas locally advanced stages are treated with definitive chemoradiotherapy (CRT), combining concurrent chemotherapy with external beam radiotherapy and brachytherapy (3, 4). More recently, immunotherapy with pembrolizumab has shown benefit in high-risk locally advanced disease and has been incorporated into treatment protocols for patients with FIGO 2014 stage IB2-IIB with node-positive disease or stage III-IVA regardless of nodal status (5). In previous research using MRI-guided adaptive brachytherapy, CRT achieves 92% 5-year local control and 87% 5-year pelvic control (6), and the addition of pembrolizumab further improves disease-free survival in selected high-risk patients (5). Nevertheless, treatment-related toxicities remain common. Radiotherapy (RT) is associated with gastrointestinal side effects such as diarrhea, nausea, and vomiting (7), as well as vaginal toxicities including vaginal atrophy, fibrosis and stenosis (8). Importantly, pelvic radiotherapy can also cause significant damage to the female reproductive organs, leading to ovarian insufficiency, uterine changes, premature menopause, and infertility (9). Chemotherapy contributes to systemic side effects such as nausea, vomiting, fatigue, and mucositis (10). In addition, immunotherapy is associated with systemic, endocrine, dermatologic, and gastrointestinal toxicities, including fatigue, fever, thyroiditis, and colitis (11, 12). These adverse effects can significantly impair patients’ quality of life both during and after treatment (13). Efforts to improve survival and reduce side effects for patients receiving therapy for locally advanced disease are essential.

Recent evidence suggests that microbial communities influence not only the development of cervical cancer, but also patients’ response to CRT and the occurrence of treatment-related side effects (14–18). In particular, the vaginal and gut microbiomes have been shown to play a role in modulating immune function and mucosal barrier integrity, with a healthy vaginal microbiome typically characterized by low diversity and a diverse gut microbiome generally considered beneficial. Microbiome-targeted interventions could therefore hypothetically mitigate severe toxicities, enhance response rates, and improve disease-free survival.

Although several reviews have addressed the role of the microbiome in cervical cancer, most have focused on a single microbial niche and were published before the recent integration of immunotherapy into the treatment of locally advanced cervical cancer. Furthermore, the complex interplay between the gut and vaginal microbiomes, their associations with treatment response and toxicity, and the translational potential of microbiome-targeted interventions have not been comprehensively evaluated. Consequently, important knowledge gaps remain regarding the microbial signatures that are consistently associated with clinical outcomes, the biological mechanisms underlying these associations, and whether modulation of the microbiome could enhance treatment efficacy while reducing treatment-related toxicity.

In this review, we aim to summarize current knowledge on the roles of the gut and vaginal microbiomes in locally advanced cervical cancer, focusing on their impact on response to CRT and treatment-induced side effects. Although the primary focus is on CRT, the effects of the microbiome on immunotherapy are also touched upon. In addition, we highlight microbiome modulation strategies and propose hypotheses to guide future studies aimed at improving treatment outcomes and quality of life for cervical cancer patients during and after therapy.

## 2 Materials and methods

**Figure 1:**
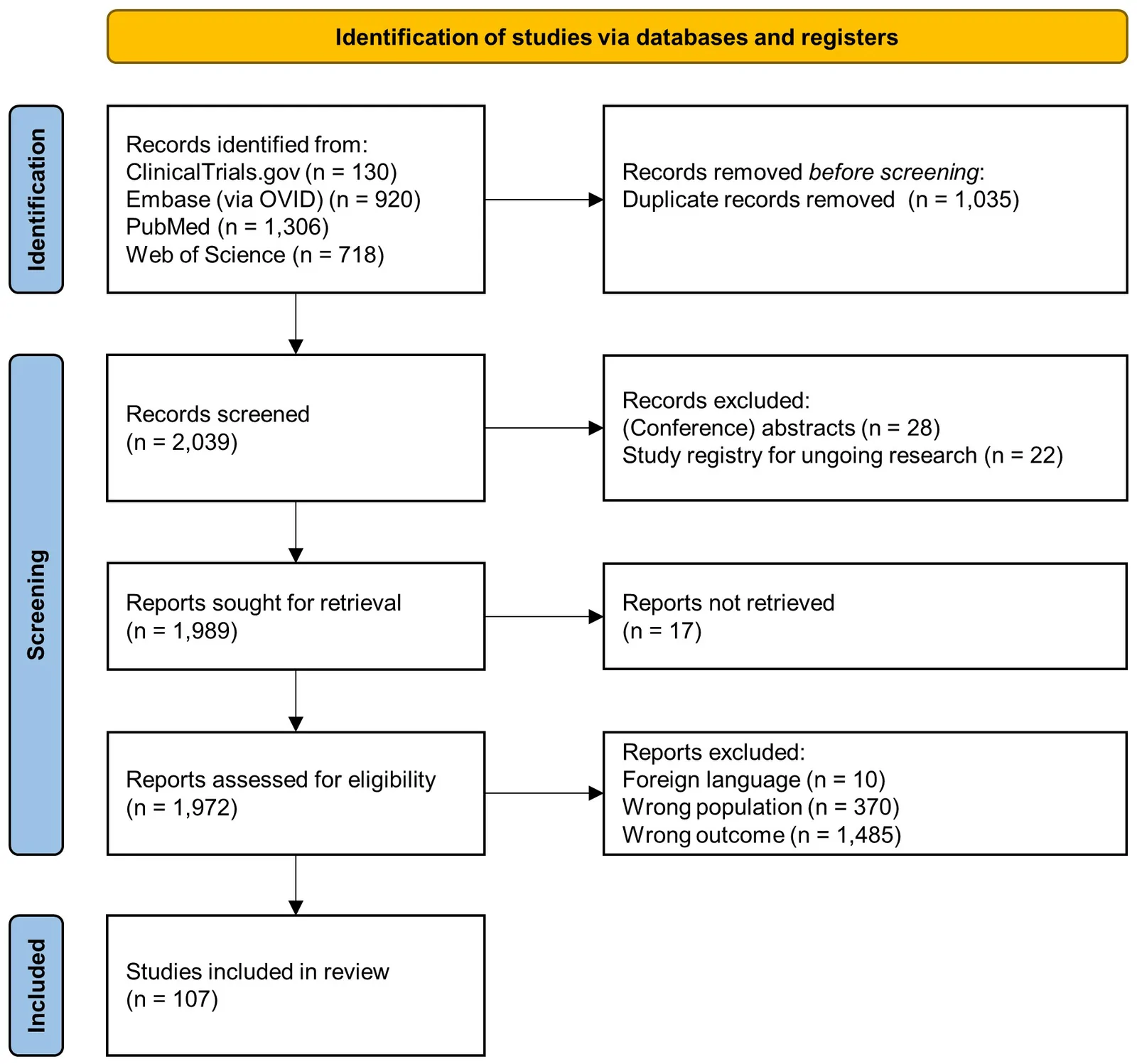
Flow diagram of study selection process.

The literature search was conducted using ClinicalTrials.gov, Embase (via OVID), PubMed, and Web of Science and was last updated on 20 April 2026 (See **Supplementary Material 1** for search strategy and further details). Two researchers (H.C.J.S. and B.J.R.B.) independently screened all records based on title and abstract. Original research articles (*in vitro*, animal, and human studies) and review articles addressing the role of the vaginal and/or gut microbiome, including their metabolites and immunological aspects, in locally advanced cervical cancer and its treatment were included. Additionally, studies investigating microbial interventions, including prebiotics and probiotics, in this context were considered. Conflicts were discussed and resolved by consensus. The remaining articles were retrieved in full text, after which additional articles were excluded if the full text was not available in English. The most relevant findings from the 107 included articles will be discussed. The study selection process is presented in a flow diagram (**Figure 1**).

## 3 Results

The human microbiome consists of multiple anatomically distinct microbial ecosystems, including the gut and vaginal microbiomes. Although these microbial communities may interact through microbial exchange, immune signaling, and metabolite-mediated pathways, they represent distinct ecological niches. Findings from one microbial compartment should therefore be interpreted cautiously and should not be directly extrapolated to another microbial environment.

### 3.1 The vaginal microbiome and cervical cancer

The vaginal microbiota describes the community of microorganisms, such as bacteria, fungi, and viruses, that naturally reside in the vagina and play a crucial role in preserving vaginal health (19).

The vaginal microbiome (VMB) refers to the collective genetic material and functional potential of these microbial communities inhabiting the vaginal environment (20). A healthy VMB is typically dominated by *Lactobacillus* species (spp.), which help maintain the integrity of the vaginal epithelial barrier through lactic acid production, antimicrobial secretion (e.g. hydrogen peroxide (H_2_O_2_), bacteriocins), and immune stimulation (21, 22). Furthermore, bioactive metabolites synthesized by vaginal lactobacilli, including exopolysaccharides, phosphorylated polysaccharides, and peptidoglycans, have demonstrated cytotoxic effects against cervical cancer cells through the inhibition of cell proliferation and the induction of apoptosis (23). The VMB can be classified into five major community state types (CSTs). CSTs I, II, III, and V are characterized by the predominance of *Lactobacillus crispatus*, *Lactobacillus gasseri*, *Lactobacillus iners*, and *Lactobacillus jensenii*, respectively. In contrast, CST IV is characterized by a low abundance of *Lactobacillus* spp. and a high diversity of anaerobic bacteria (23, 24). More recently, these CSTs have been further subdivided into a total of 13 subtypes based on the relative abundance of the dominant species. This refined classification enables a better understanding of VMB dynamics than the original five CSTs. Nevertheless, in this paper, we focus on the major CSTs, as these are currently the most commonly used classifications in the literature (25). Shifts toward a dysbiotic VMB, characterized by high diversity and an overgrowth of bacterial vaginosis (BV)-associated bacteria (*Gardnerella, Atopobium, Prevotella*, and *Sneathia* spp.), have been associated with cervical carcinogenesis (21, 23). However, it is also plausible that a necrotic and hypoxic tumor microenvironment may further promote the growth of anaerobic species, potentially at the expense of lactobacilli (26).

Recent studies have further characterized these microbial shifts, revealing distinct alterations in the VMB of patients with cervical carcinoma. Microbial richness and biodiversity (α-diversity) progressively increase from women with normal cytology to those with invasive cancer, accompanied by significant shifts in microbial community structure (β-diversity) and enrichment of taxa within the phylum of Pseudomonadota (previously Proteobacteria) (16–18, 23). Specifically, the abundance of *Lactobacillus* spp. decreases and CST IV becomes more prevalent in cervical cancer patients (16, 18) with enrichment of various dysbiosis-associated taxa and opportunistic pathogens (*Streptococcus*, *Peptostreptococcus*, *Enterococcus*, *Escherichia–Shigella*, *Porphyromonas*, *Sneathia*, and *Anaerococcus* spp.) (17, 18). Invasive cervical cancer is particularly associated with *Fusobacterium* spp., which correlate with elevated tissue levels of immunosuppressive cytokines, indicative of an immunosuppressive tumor microenvironment (TME) (23). However, it remains unclear whether the immunosuppressive TME precedes or arises as a consequence of microbial dysbiosis.

As the VMB shifts from a *Lactobacillus*-dominant environment, in which lactic acid dominates, to one enriched in anaerobic taxa, the production of vaginal short chain fatty acids (SCFA) such as acetate, propionate, and butyrate increases (23, 27, 28). However, the biological effects of SCFAs are highly context-dependent. In the gastrointestinal tract, SCFAs, particularly butyrate, generally support epithelial barrier integrity and immune homeostasis. In contrast, within a bacterial vaginosis-like vaginal environment, increased SCFA concentrations occur together with reduced lactic acid production, elevated vaginal pH, and enrichment of anaerobic taxa (18, 23). This metabolic shift is associated with the loss of the protective effects of a *Lactobacillus*-dominant environment, including reduced pathogen inhibition and altered immune regulation, thereby contributing to a pro-inflammatory state and impaired epithelial barrier function (21, 23, 29, 30).

Dysbiotic cervicovaginal communities are not only linked to the production of vaginal SCFAs, but also directly to the activation of local immune cells. Whereas acute inflammation is considered beneficial for HPV clearance, chronic inflammation is associated with tumorigenesis (23). Immune evasion mechanisms are central to this transition (31, 32). In the vaginal microenvironment, CSTs III and IV are associated with increased expression of pro-inflammatory cytokines (23), whereas *Lactobacillus* spp. are associated with the activation of immune factors that support tissue repair and exert anti-inflammatory effects (23, 33). The intratumoral microbiome can activate inflammatory signaling cascades through the engagement of pattern recognition receptors (PRRs), such as TLRs, within the TME. This activation promotes the recruitment of immune cells and stimulates the production of pro-inflammatory mediators that promote a persistent inflammatory milieu. Microbial stress and tissue damage further enhance the release of reactive oxygen species (ROS), leading to oxidative DNA damage and genomic instability. Acute inflammation is typically a self-limiting process consisting of an initiation and resolution phase that restores immune homeostasis. In contrast, chronic inflammation is characterized by the absence of a complete resolution phase due to sustained exposure to inflammatory factors (34). Over time, this chronic inflammatory environment shifts toward an immunosuppressive state that facilitates tumor progression and inhibits antitumor immune responses (31, 32, 34). However, findings from intratumoral microbiome studies should be interpreted with caution. It remains unclear whether detected microbiota actively contribute to tumor biology or merely colonize tumor tissues. Moreover, concerns regarding contamination and the frequent detection of only a limited number of microbial species, potentially insufficient to constitute a true microbiome, further complicate interpretation (31, 35).

### 3.2 The gut microbiome and cervical cancer

Like the VMB, the gut microbiome is the community of microorganisms and its theatre of activity inhabiting the gastrointestinal tract. A healthy human gut microbiome is dominated by five bacterial phyla: Actinomycetota (previously Actinobacteria), Bacteroidota (previously Bacteroidetes), Bacillota (previously Firmicutes), Fusobacteriota (previously Fusobacteria), and Pseudomonadota, which play critical roles in metabolizing dietary compounds, synthesizing vitamins, preserving intestinal barrier integrity, and regulating immune responses (21, 22, 36, 37). In an eubiotic state, a diverse gut microbiome interacts harmoniously with the host immune system to preserve intestinal homeostasis and to prevent pathogenic colonization. Disruption of this balance, known as gut microbial dysbiosis, results in reduced microbial diversity and a decline in beneficial commensal bacteria, contributing to disease development (36).

Studies comparing the gut microbiome of cervical cancer patients and healthy individuals reveal increased dysbiosis in cervical cancer patients, characterized by depletion of protective commensals like Bacillota (including butyrate-producing *Clostridium* and *Ruminococcus* spp.) and Fusobacteriota, limiting the production of anti-inflammatory metabolites, and increases in Actinomycetota, Bacteroidota, and opportunists (*Prevotella, Porphyromonas*, and *Dialister*) (14, 21, 38, 39). However, findings at the phylum level should be interpreted with caution, as these taxonomic groups encompass both bacteria associated with gut homeostasis and those linked to dysbiosis.

The gut microbiome influences host physiology primarily through the production of microbial metabolites, which function as signalling molecules and metabolic substrates, exerting both pro- and anti-carcinogenic effects depending on the environmental context (40). In particular, symbiotic bacteria, including *Lactobacillus* and *Bifidobacterium* spp., are crucial for maintaining microbial homeostasis by producing metabolites such as lactate and SCFAs, which regulate immune responses and support epithelial repair (36). SCFAs are primarily generated through microbial fermentation of dietary fibres and are produced by a variety of taxa across different phyla (40, 41). The major SCFAs, acetate, propionate, and butyrate, are produced by distinct but overlapping microbial communities. Acetate is produced by, amongst others, *Akkermansia muciniphila*, *Bacteroides* spp., and *Bifidobacterium* spp. whereas butyrate is mainly synthesized by anaerobic bacteria such as *Faecalibacterium* spp. and *Roseburia* spp (41)., as well as by cross-feeding species like *Eubacterium hallii*, which utilize intermediates such as lactate and acetate (42). Propionate is largely produced by *Bacteroides* spp., often via lactate-dependent pathways (41). This highlights the metabolic interdependence within the gut microbiome, where the production of specific metabolites relies on interactions between microbial species.

SCFAs are absorbed by the epithelial cells of the colon. Most butyrate and a substantial portion of propionate are metabolized by colonocytes and the liver, whereas acetate more readily escapes into the systemic circulation. Although circulating SCFA concentrations are lower than in the colon epithelium, they are believed to be sufficiently high to affect host cells (41, 43). The mentioned SCFAs, particularly butyrate, inhibit cancer cell proliferation and promote apoptosis, partly through the reduction of pro-inflammatory cytokines (36, 44). In addition, butyrate supports intestinal barrier integrity, thereby limiting the translocation of bacteria and toxins into the bloodstream and TME (21, 45, 46). By maintaining a low intestinal pH, SCFAs further promote the growth of beneficial bacteria and inhibit colonization by pathogenic species (21). Butyrate and propionate act as inhibitors of histone deacetylase (HDAC), an enzyme crucial for various cellular processes including proliferation, differentiation, and apoptosis. As HDAC-inhibitors, these metabolites influence innate and adaptive immune responses by suppressing NF-κB activity, enhancing the expression of Toll-like receptors (TLR) and antimicrobial peptides, and promoting the production of anti-inflammatory cytokines, and supporting epithelial repair (41, 47, 48), while also promoting regulatory T cell (Treg) differentiation through increased histone acetylation and upregulation of FOXP3 (49). In contrast, acetate exhibits weaker HDAC inhibitory activity but can modulate immune responses by influencing reactive oxygen species (ROS) production, TLR signaling, and dendritic cell function (41). Moreover, SCFAs modulate the activation of CD8+ cytotoxic T lymphocytes to support anti-tumor immunity, with such infiltrates linked to improved survival in HPV-related tumors (50).

In addition to SCFAs, a broad range of microbial-derived metabolites contribute to host-microbiome interactions and may influence cervical carcinogenesis. Key metabolite groups include secondary bile acids and tryptophan-derived compounds, generated through microbial metabolism of dietary components such as fibers and aromatic amino acids, as well as host-derived substrates including primary bile acids (49, 51). Members of the phylum Bacillota can convert primary bile acids into secondary bile acids such as deoxycholic acid and lithocholic acid, which exert context- and concentration-dependent effects on inflammation and tumor biology (51). High-fat diets may increase bile secretion and elevate levels of these metabolites, contributing to a pro-inflammatory, tumor-supportive environment, whereas fiber-rich diets promote the production of metabolites associated with beneficial effects, including butyrate (49).

Another key pathway linking the gut microbiome to immune regulation is tryptophan metabolism. Indole derivatives, produced by *Lactobacillus* and *Bifidobacterium* spp., activate the aryl hydrocarbon receptor (AhR), supporting immune homeostasis and epithelial barrier integrity (49, 51). In contrast, upregulation of the kynurenine pathway via indoleamine 2,3-dioxygenase (IDO1) and tryptophan 2,3-dioxygenase (TDO) leads to tryptophan depletion and accumulation of immunosuppressive metabolites, resulting in T cell anergy and a tumorigenic environment (49, 51).

In addition to these metabolite-mediated effects, microbial ligands further shape immune responses. Lipopolysaccharides and peptidoglycans can stimulate TLRs on enterocytes and dendritic cells, mediating a T-cell response against tumor cells. The commensal bacterium *Bacteroides fragilis* produces Polysaccharide A, which is recognized by TLR-2. This interaction induces Tregs and stimulates interleukin (IL)-10 production, thereby triggering an anti-inflammatory intestinal environment and maintaining immune homeostasis (21, 46, 48, 52, 53). In parallel, other microbial products, including exopolysaccharides, have demonstrated anti-tumor effects *in vitro*, including inhibition of HeLa cell proliferation and modulation of apoptotic gene expression (54).

Antimicrobial peptides (AMPs) further contribute to these interactions in a concentration-dependent manner, inducing tumor cell apoptosis and modulating immune responses in lower doses, while disrupting cellular membranes at higher concentrations. Additionally, AMPs contribute to maintaining *Lactobacillus* dominance, thereby reinforcing a protective microbial environment (55). Moreover, bacterial products can stimulate immune cells to produce interferon (IFN)-γ, promoting neutrophil survival and maturation, thereby enhancing immune-mediated anti-tumor response (53). In cervical cancer patients, higher gut microbial diversity was associated with increased tumoral infiltration of CD4+ T cells, suggesting that a more diverse, healthy, gut microbiome may support a stronger immune-mediated response (15).

Concluding, disturbance of the gut microbiome is thought to promote chronic inflammatory responses. Persistent inflammatory signaling, driven by ROS, cytokines, and chemokines, supports cancer cell proliferation, invasion, migration, and immune evasion (21, 22, 56). These findings suggest that alterations in specific microbial taxa and their metabolites may contribute to cervical carcinogenesis, potentially through mechanisms involving the transition from acute to chronic immune response, as well as impaired intestinal barrier integrity. However, as previously discussed, the question remains whether dysbiosis-driven immune modulation or immune-driven microbial shifts constitute the primary driver of these associations. Moreover, these effects are context-dependent, as bacteria and their metabolites can exert both pro- and anti-inflammatory functions. Consequently, attributing specific functional effects to individual bacterial strains remains challenging.

**Table 1** summarizes the key metabolite-mediated mechanisms in (cervical) cancer.

**Table 1:** Key microbiome-derived metabolites and their immunological and biological effects.

Microbial metabolite	Microbial source	Metabolite-mediated effects	References
Butyrate	Mainly produced by anaerobic bacteria including *Faecalibacterium* spp., *Roseburia* spp., and *Eubacterium hallii*	- Inhibits cancer cell proliferation - Promotes apoptosis - Reduces pro-inflammatory cytokine production - Supports intestinal barrier integrity - Inhibits histone deacetylase (HDAC), thereby modulating innate and adaptive immune responses and promoting epithelial repair	(21, 36, 41, 42, 44–48)
Propionate	Mainly produced by *Bacteroides* spp.	- Inhibits HDAC, thereby modulating innate and adaptive immune responses and promoting epithelial repair	(41, 47, 48)
Acetate	Produced by *Akkermansia muciniphila, Bacteroides* spp., and *Bifidobacterium* spp.	- Exhibits weak HDAC inhibitory activity - Modulates immune responses by influencing reactive oxygen species (ROS) production	(41)
Secondary bile acids (e.g. deoxycholic acid, lithocholic acid)	Produced through conversion of primary bile acids by members of the phylum Bacillota	- Elevated levels of secondary bile acids may contribute to a pro-inflammatory, tumor-supportive environment	(49, 51)
Tryptophan-derived metabolites (e.g. indoles)	Produced by *Lactobacillus* spp. and *Bifidobacterium* spp.	- Indole derivatives activate the aryl hydrocarbon receptor (AhR), supporting immune homeostasis and epithelial barrier integrity - Tryptophan depletion leads to accumulation of immunosuppressive metabolites, resulting in T cell anergy and a tumorigenic environment	(49, 51)
Microbial ligands	Produced by various bacteria (e.g. polysaccharide A by *Bacteroides fragilis*)	- Lipopolysaccharides and peptidoglycans activate toll-like receptors (TLRs) on enterocytes and dendritic cells, thereby promoting T-cell-mediated immune responses against tumor cells - Polysaccharide A triggers an anti-inflammatory intestinal environment and maintains immune homeostasis	(21, 46, 48, 52, 53)

(This table summarizes the key microbiome-derived metabolites, their principal microbial sources, and proposed immunological and biological effects).

### 3.3 The crosstalk between the vaginal microbiome and the gut microbiome

The gut and vaginal microbiomes are distinct yet interconnected ecosystems that can influence each other’s composition. The gut-vaginal axis refers to the bidirectional interactions between these microbial communities. The close anatomical proximity of the vagina and the gut allows for bacterial transmission along this axis. Evidence for this microbial trafficking has been demonstrated by the detection of probiotic strains in the vagina following oral administration, indicating that certain gut-resident bacteria are capable of colonizing the vaginal niche. Correlative microbiome analyses further support this connection by identifying BV-associated taxa in both the vaginal and rectal microbiota of pregnant women. This suggests that microbial exchange can occur via direct transfer, and indirect pathways, including immune- or metabolite-mediated modulation of the microbiome (28, 57).

Functional interactions between the gut and vaginal ecosystem occur not only through microbial migration but also via shared metabolic pathways. Following intestinal production, SCFAs are largely absorbed by intestinal epithelial cells and can enter systemic circulation, where they exert immunomodulatory effects on distant tissues (46, 57). It can be hypothesized that gut-derived metabolites may contribute to a gut-reproductive tract axis. However, direct evidence demonstrating that intestinally derived SCFAs reach the vaginal space at biologically relevant concentrations is lacking. Therefore, potential effects of gut-derived SCFAs on the vaginal environment should currently be considered a proposed mechanism rather than an established pathway (30). Moreover, estrogen plays a fundamental role in mediating the effect of gut microbiota on reproductive tract bacteria. Gut bacteria metabolize estrogen, thereby influencing systemic estrogen levels. Specific bacterial taxa, such as *Clostridia* and *Ruminococcacaeae*, are particularly linked to estrogen production (36). This interaction is known as the estrobolome. The liver secretes conjugated estrogens, which are then deconjugated in the gastrointestinal tract by certain gut bacteria that produce β-glucuronidase. This allows active estrogens to be reabsorbed into circulation via the enterohepatic cycle, thus contributing to the maintenance of hormonal balance. However, excessive β-glucuronidase activity, often associated with dysbiosis, leads to increased reabsorption of estrogens, resulting in elevated circulating estrogen levels, which can have oncogenic potential (21, 36, 57). Unconjugated estrogen then enters the systemic circulation and is transported to peripheral organs, including the female lower reproductive tract, where it binds to estrogen receptors and activates downstream cellular signaling pathways. This, in turn, stimulates glycogen synthesis in vaginal epithelial cells, providing an essential energy source for *Lactobacillus* spp., which dominate the healthy vaginal microbiome. Consequently, the abundance and activity of estrogen-metabolizing bacteria in the gut can profoundly influence the composition and stability of the vaginal microbial community. This estrogen-dependent regulation is especially evident in postmenopausal women, in whom declining estrogen levels cause a reduction in protective *Lactobacillus* populations, eventually leading to vaginal dysbiosis (57). These menopause-associated alterations in the vaginal microbiome may contribute to an increased risk of cervical cancer development (58).

### 3.4 The interplay between the microbiome and chemoradiotherapy for locally advanced cervical cancer

As mentioned, treatment depends on disease stage: early-stage options include radiotherapy or surgery, whereas locally advanced cervical cancer typically requires CRT, recently when indicated with the addition of pembrolizumab (5, 59). Therapeutic outcomes are influenced by a complex interplay of factors, among which the human microbiome is gaining interest. Increasing evidence underscores the pivotal role of the microbiome in modulating both treatment toxicity and efficacy (60).

As radiotherapy disturbs healthy mucosal, stromal, endothelial, and glandular cells it is associated with side effects, including vaginal radiation toxicity, urinary tract toxicity and gastrointestinal radiation toxicity. These toxicities can be categorized into acute and late effects. Acute vaginal toxicity includes inflammation, dryness, swelling, hyperemia and increased vaginal discharge, while late effects involve loss of capillaries, reduced endothelial cell proliferation, and increased collagen production, leading to mucosal atrophy, bleeding and vaginal shortening and narrowing. Collectively, these adverse effects can severely impact quality of life (61, 62). Radiation-induced cystitis can also occur due to unintentional irradiation of the bladder during cervical cancer treatment (37). As the literature on the role of the microbiome in urinary tract toxicity is limited, it is not a focus of this review. Gastrointestinal toxicity such as bleeding and diarrhea occur in up to 90% of patients receiving pelvic radiotherapy (60) and one-third of the gynecological patients treated with pelvic radiotherapy are, in the long term, left with significant gastrointestinal dysfunction (63). Pelvic radiotherapy impairs the renewal capacity of the intestinal epithelium. During treatment, a study of rectal biopsies reported more pronounced epithelial atrophy, acute cryptitis, crypt abscesses, and stromal inflammation (64). Pelvic radiotherapy also induces vascular damage, which may result in chronic rectal symptoms due to telangiectasia and consequent rectal bleeding (65). Diarrhea may result from several mechanisms associated with inflammatory changes in the intestine, including increased gastrointestinal motility (potentially induced by endotoxins released by pathogenic bacteria), reduced bile acid reabsorption with consequent impairment of lipid digestion and absorption, and impaired tissue renewal (66).

Additionally, radiotherapy can alter both the vaginal and gut microbiome composition, although these microbial ecosystems differ substantially in their baseline composition, metabolic functions, and interactions with host tissues. Therefore, treatment-induced microbial alterations should be interpreted within the specific anatomical and biological context of each microbial compartment. In the gut, these changes are characterized by reduced microbial diversity and a shift toward communities enriched in opportunistic bacterial pathogens (23, 61). Greater reductions in gut microbial diversity have been associated with worse patient-reported and clinically assessed gastrointestinal outcomes in cervical cancer patients (63). Moreover, microbiota are thought to influence treatment outcomes by modulating immune responses and affecting enzyme activity and drug metabolism (67). While such interactions can be beneficial, they may also lead to reduced effectiveness, increased toxicity, and the development of treatment resistance (67). Administration of chemotherapeutic agents can further alter microbial composition, leading to reduced gut microbial α-diversity and an increased abundance of pro-inflammatory species causing adverse effects, like diarrhea and mucositis (60).

However, the question remains: does dysbiosis arise prior to CRT or as a consequence of it? Moreover, are the adverse effects primarily driven by CRT itself, by a less beneficial baseline microbiome, or by a combination of both?

#### 3.4.1 The impact of chemoradiotherapy on the vaginal microbiome

Recent studies have explored alterations in the VMB of patients with gynecologic cancers, including cervical cancer, in response to cancer therapy. Overall, these studies report a shift toward higher microbial diversity and a depletion of *Lactobacillus* spp., often accompanied by an increased abundance of anaerobic and bacterial vaginosis-associated taxa, both during and after treatment.

Cervical cancer patients showed significantly increased α-diversity post-radiotherapy, with increased abundances of dysbiosis-associated taxa (*Mobiluncus*, *Atopobium*, and *Prevotella*) and reduced abundances of *Lactobacillus, Gardnerella*, and *Peptostreptococcus* (23, 68). Similarly, Ivanov et al. observed that post-treatment, *L. iners* was increased, accompanied by persistent enrichment of bacterial vaginosis and aerobic vaginitis associated bacteria including *Mobiluncus, Prevotella, Veillonella, Megasphaera*, and *Sneathia* (16).

Other studies support these findings. Tortelli et al. reported dominance of anaerobes *Prevotella, Porphyromonas, Peptoniphilus, Anaerococcus*, and *Fusobacterium* in postmenopausal women with locally advanced cervical cancer receiving chemoradiation (n=26), both before and after CRT, with *Lactobacillus* notably absent in most patients. Alpha diversity did not significantly change over time (69). In contrast, Tsementzi et al. found significantly higher α-diversity in gynecologic cancer patients (n=65, including 29 with cervical cancer) post-radiotherapy as compared to pre-treatment samples, along with increased abundances of taxa associated with dysbiosis (*Prevotella, Pseudomonas*, and members of the Lachnospiraceae family) (70). Longitudinal analysis by Wang et al. (n=125) showed Bacillota, Pseudomonadota, and Bacteroidota as dominant phyla pre- and post-CRT. Bacteroidota and Bacillota decreased, while Pseudomonadota increased throughout treatment (71). Consistently, Bi et al. showed that vaginal radiotherapy resulted in an increased relative abundance of Pseudomonadota and a concomitant decrease in Bacillota in gynaecological cancer patients (n=54) (61). Collectively, these studies suggest that radiotherapy and CRT are associated with a shift toward a dysbiotic vaginal microbiome, often characterized by a depletion of *Lactobacillus* spp. and enrichment of anaerobic taxa, while reported changes in α-diversity remain inconsistent across studies. These compositional changes may have functional consequences, as depletion of *Lactobacillus* spp. reduces lactic acid production and may impair vaginal mucosal protection, thereby increasing susceptibility to inflammation, opportunistic pathogen colonization, and radiation-induced tissue injury (72, 73). However, whether these microbial alterations directly contribute to toxicity or primarily reflect radiation-induced epithelial damage remains unclear.

#### 3.4.2 The impact of the vaginal microbiome on chemoradiotherapy outcomes and toxicity

Recent evidence highlights the emerging role of the vaginal microbiome in modulating both the efficacy and toxicity of chemoradiotherapy. In general, dysbiosis-associated bacteria are associated with poorer clinical outcomes and increased treatment-related toxicity. Tortelli et al. found no difference in bacterial abundance between patients who developed disease recurrence and those who did not (n=26), however although not statistically significant, greater pre-treatment abundances of dysbiosis-associated *Fusobacterium* were observed among patients who later had cancer recurrence (69). In previous research, intratumoral *Fusobacterium* was associated with more advanced stages of cervical cancer and poor progression free survival (74). Importantly, findings from intratumoral studies should not be directly extrapolated to the vaginal environment, as these represent distinct microbial compartments with different host interactions. A study investigating alterations in de vaginal microbiome following concurrent CRT in 125 cervical cancer patients found that VMB stability could be of importance. Stability refers to the likelihood of disturbance and changes in VMB composition. This stability was more altered in patients with a local or distant recurrence following initial treatment. Additionally, in the recurrence group, *L. iners* was mainly enriched pre-CRT. This could indicate that *L. iners* could serve as a predictor of cancer recurrence after CRT (71). Another study (n=26) showed higher α-diversity and enrichment of Pseudomonadota and Bacteroidota in non-responders (patients with stable disease or progression), suggesting its potential in predicting the response to treatment (17).

Dou et al. (n=36) found that cervical cancer patients with superior response (SR: tumor volume reduction rate (TVRR) >66.7%; n=26) to RT had lower tumor microbial α-diversity and higher abundances of Bifidobacteriaceae, a family that includes both beneficial taxa, such as *Bifidobacterium*, and potentially pathogenic members, including *Gardnerella.* There were also increased levels of Beijerinkiaceae and Orbaceae, whose roles in the tumor or vaginal microbiome remain unclear. In contrast, inferior responders (IR: TVRR <66.7%; n=10) were enriched with potentially pathogenic taxa, including Sphingomonadaceae, Caulobacteraceae, Pseudonocardiaceae, and Rhizobiaceae (75). The SR group showed higher counts of CD8+ T cells and GzmB+ cells, suggesting that the increased abundance of Bifidobacteriaceae and the lower α-diversity within SR tumors may facilitate the recruitment and activation of CD8+ T cells in the tumor microenvironment, thereby enhancing the anti-tumor immune response and improving treatment outcomes. This is further supported by the prediction that progression-free survival and overall survival are more favorable in patients with a lower microbial biodiversity and higher abundance of Bifidobacteriaceae. Previous studies have suggested that *Bifidobacterium* has anti-cancer effects by modulating the immune response, particularly through the enhancement of DC activation, which promotes CD8+ T cell priming and accumulation within the tumor microenvironment, ultimately contributing to improved anti-tumor response. This favorable immunological milieu, when combined with external beam radiotherapy (EBRT), may act synergistically to enhance tumor cell eradication, thereby improving therapeutic outcomes in patients with cervical cancer (75).

Radiotherapy induces epithelial damage, causing severe toxicities, including vulvovaginal atrophy, vaginal stenosis, and pelvic pain (23). Bi et al. (n=54 gynecologic cancer patients) found an increase in pathobiont *Brevundimonas* and other Gram-negative non-fermenting bacteria in patients with level 2 vaginal radiation injury (61). Post-radiotherapy enrichment of urease-producing Gram-negative bacteria (*Brevundimonas*, *Rhizobium*, and *Ralstonia*) promotes ureolytic metabolism, which converts urea into ammonia and carbon dioxide. This process is associated with mucosal inflammation, including gastric and intestinal mucositis, and may similarly trigger inflammatory and cytotoxic effects on the vaginal mucosa (61). Additionally, radiotherapy was linked to a decrease in fermentative capacity. Reduced production of fermentation-related enzymes likely diminishes the synthesis of lactic acid and SCFAs. Collectively, these findings suggest that radiotherapy-induced alterations in the structure and function of the VMB may contribute to subsequent vaginal tissue damage (61).

#### 3.4.3 The impact of chemoradiotherapy on the gut microbiome

Several studies have investigated the impact of RT and CRT on the gut microbiome in cervical cancer patients. Overall, CRT is consistently associated with a decrease in gut microbial diversity and shifts in taxonomic composition to communities associated with dysbiosis.

Longitudinal studies show that gut microbial richness and diversity decline during CRT but may partially recover post-treatment (38, 76, 77). El Alam et al. observed decreased richness and diversity by week 5 of CRT (n=58), characterized by a depletion of beneficial taxa, particularly within the class Clostridia (including *Faecalibacterium* and *Ezakiella*) and enrichment of potentially pathogenic taxa such as the phylum Pseudomonadota (including *Haemophilus*). These changes normalized post-CRT, although the abundance of Bacteroidota was more persistently decreased (76). These longitudinal observations suggest that CRT-induced microbiome alterations are dynamic rather than permanent. The transient depletion of SCFA-producing bacteria may be particularly relevant, as reduced butyrate availability could impair epithelial barrier integrity and promote inflammation. However, whether microbiome recovery contributes to restoration of intestinal function or merely reflects cessation of treatment remains unknown. It could be hypothesized that CRT can initiate changes in the gut epithelium and mucus barrier, which may facilitate pathogenic bacterial overgrowth, resulting in colonization of bacterial populations tolerant to radiation-induced stress. This may impair immune maturation and activation and treatment response. Higher CRT doses may further induce overgrowth of radiation-tolerant microbes. The gut microbial community remained altered three months post-treatment, suggesting incomplete microbiome recovery. This sustained dysbiosis likely reflects the transient predominance of potentially pathogenic bacteria and loss of beneficial taxa during CRT, followed by compensatory expansion of Bacteroidota to restore ecological balance (76). Nam et al. reported progressive declines in Bacillota and *Fusobacterium* during RT, with gradual post-treatment recovery (n=9) (38). Mitra et al. observed a progressive reduction in α-diversity during treatment, with a specific decline in Clostridiales (n=35) (78). Additionally, Klimov-Kravtchenko et al. reported a depletion of SCFA-producing bacteria following CRT in cervical cancer patients (n=22) compared to untreated patients (n=27) (79).

#### 3.4.4 The impact of the gut microbiome on chemoradiotherapy outcomes and toxicity

Emerging evidence highlights the critical role of the microbiome in modulating radiotherapy toxicity and efficacy.

Several studies have explored associations between the microbiome and treatment response. Sims et al. (n=55) observed that greater baseline gut microbiome diversity was associated with improved treatment response. Specifically, a higher Shannon Diversity Index correlated with longer median recurrence-free survival (RFS) and overall survival (OS). Short-term survivors were enriched in *Porphyromonas, Dialister*, and Porphyromonadaceae, whereas long-term survivors had higher abundances of *Escherichia/Shigella*, Enterobacteriaceae, and Enterobacteriales (15). These findings suggest that baseline microbiome composition may have predictive value; however, microbial diversity alone is unlikely to represent a sufficient biomarker, as taxonomic composition, microbial functionality, and host-related factors may collectively determine treatment response.

Similarly, Colbert et al. investigated the gut microbiome of 101 patients with locally advanced cervical cancer undergoing chemoradiation. In a pilot subset, *L. iners* was significantly associated with poorer responses to CRT as an increased baseline abundance correlated with decreased RFS and OS, whereas the presence of Pseudomonadota, Gammaproteobacteria, and Actinomycetota were associated with better treatment outcomes. Relative abundances of *L. iners* did not significantly change during or after CRT. At baseline, presence of *L. iners* was associated with higher infiltration of CD4+ and CD8+ T cells. However, these levels decreased during CRT regardless of *L. iners* status. To investigate whether *L. iners* contributes to radiation resistance, patient-derived organoids (PDOs) were developed. PDOs co-cultured with CC-*L. iners* showed more aggressive growth and radiation resistance compared with controls, whereas *L. crispatus* did not induce resistance. Metabolomic profiling revealed that CC-*L. iners* primarily produced L-lactate *in vitro*, while *L. crispatus* produced D-lactate. Pre-treatment of cervical cancer cell lines with lactate isoforms demonstrated that L-lactate, but not D-lactate, induced treatment resistance, consistent with the effect observed for *L. iners* (26). Elevated levels of L-lactate in the tumor microenvironment contribute to an immunosuppressive milieu. High L-lactate concentrations inhibit T-cell activation and proliferation, promoting T-cell anergy and reducing the effectiveness of cytotoxic lymphocyte infiltration. Consequently, cancer cells can escape immune surveillance and persist (80). The elimination of L-lactate producing bacteria could possibly enhance therapeutic responses (81).

In parallel with these effects on treatment efficacy, radiotherapy also induces alterations in gut microbial composition that are linked to toxicity. Radiotherapy reduces gut microbial diversity, especially in patients with diarrhea (60). In these patients, there is an increase in the abundance of *Bacteroides, Dialister* and *Veillonella* and a decrease of *Clostridium, Faecalibacterium, Oscillibacter, Parabacteroides*, and *Prevotella* (60). Patients experiencing minimal gastrointestinal toxicity were enriched in Bacillota throughout chemoradiation therapy (78). He et al. demonstrated that abdominal irradiation results in a marked reduction in *Akkermansia muciniphila* abundance, which is negatively correlated with the duration of clinical diarrhea in patients. Moreover, propionic acid, a short-chain fatty acid secreted by this microbe, was shown to exert a protective effect against irradiation-induced injury by promoting mucus production and upregulating tight junction proteins (77). While *A. muciniphila* plays a key role in maintaining gut barrier integrity, its effects can potentially be context- and dose-dependent. In animal models, excessive colonization has been shown to disrupt epithelial integrity, leading to increased systemic inflammation and heightened susceptibility to tumorigenesis. Collectively, these findings highlight the dual and context-dependent role of specific microbial taxa and underscore the importance of functional validation to identify strain-specific therapeutic potential (82, 83).

Specific microbial species have been linked to radiation-induced enteritis (RE). Ma et al. reported that a higher Bacteroidota/Bacillota ratio, decreased abundance of beneficial *Blautia*, and increased opportunistic *Escherichia-Shigella* were associated with RE and severe acute radiation enteritis (SARE) (n=65), while findings for *Faecalibacterium* were inconsistent (84). Similarly, Wang et al. (n= 18: 10 RE patients and 8 non-RE patients) reported gut dysbiosis in patients who developed RE, characterized by a significant reduction in α-diversity, increased Pseudomonadota and *Gammaproteobacteria*, decreased *Bacteroides*, and pre-RT enrichment of *Coprococcus.* α-diversity further declined in the severe toxicity group compared with the mild toxicity group, suggesting a gradual microbial response to radiation exposure (85).

Moreover, Mitra et al. observed that higher baseline α-diversity was associated with more favorable bowel scores. Although no significant differences in microbial community structure (β-diversity) were detected between high and low toxicity groups at baseline, distinct profiles were seen at week 5 of treatment. Patients with high toxicity had increased relative abundances of *Phascolarctobacterium*, Lachnospiraceae*, Veillonella*, Erysipelotrichaceae, and Fecalitalea, whereas the low toxicity group retained higher levels of Clostridiales (78). An overgrowth of potentially pathogenic taxa in high toxicity patients may promote the production of toxic metabolites, damage the epithelial barrier, increase intestinal permeability, and facilitate the persistence of pathogenic microbes, thereby altering the gut environment and driving dysbiosis (78). Together, these findings suggest that radiation-induced toxicity is not solely determined by microbial composition at a single time point, but may reflect dynamic changes in barrier function and metabolite production throughout treatment. The relevance of microbial metabolites was further highlighted by Shen et al., who reported increased levels of primary bile acids and decreased concentrations of secondary bile acids in cervical cancer patients with chronic RE (n=20) compared to both untreated patients (n=20) and patients without chronic RE after radical radiotherapy (n=20) (86).

Overall, longitudinal studies across both the gut and vaginal microbiome indicate that CRT induces measurable disruptions in both the gut and vaginal microbiomes, characterized by reduced microbial stability and loss of potentially beneficial taxa. However, current evidence does not yet establish whether these microbial alterations are drivers of treatment toxicity and efficacy or consequences of treatment-induced tissue injury.

**Table 2** provides a summary of the roles of several bacterial taxa.

**Table 2:** Vaginal and gut microbiome shifts during CRT for locally advanced cervical cancer.

Microbial taxa	Role	Microbial patterns in cervical cancer patients (pre-, during and post-treatment)	References
Vaginal Microbiome			
*Lactobacillus crispatus* (CST I) *Lactobacillus gasseri* (CST II) *Lactobacillus jensenii* (CST V)	Beneficial	↓ throughout CRT; maintain low pH, epithelial barrier, antimicrobials, immune support.	(21, 22, 71)
*Lactobacillus iners* (CST III)	Dysbiosis-associated	↑ pre-CRT in recurrence patients; enriched post-CRT.	(16, 71)
CST IV/dysbiosis-associated bacteria (*Gardnerella vaginalis*, *Atopobium vaginae*, *Sneathia amnii*, *Prevotella* spp., *Fusobacterium* spp.)	Dysbiosis-associated	↑ pre- and post-CRT; promote inflammation, cervical carcinogenesis, associated with poor progression free survival and recurrence.	(16–18, 21, 23, 69, 71, 74)
Gut Microbiome			
Actinomycetota (*Bifidobacterium*)	Beneficial	↓ pre-CRT; SCFA production preserves epithelial barrier.	(21, 36)
Bacteroidota (*Bacteroides fragilis*)	Beneficial	↓ during CRT and in patients with gastrointestinal side effects; induces anti-inflammatory cytokines.	(48, 76, 85)
Bacillota (*Clostridium, Ruminococcus, Faecalibacterium, Lactobacillus*)	Beneficial	↓ pre-, during, and post-CRT and in patients with gastrointestinal side effects. ↑ in low toxicity group; SCFA production preserves epithelial barrier.	(14, 21, 36, 38, 39, 60, 84, 114)
Fusobacteriota	Beneficial	↓ pre- and during CRT.	(14, 21, 38, 39)
Pseudomonadota	Dysbiosis-associated	↑ during CRT and in patients with gastrointestinal side effects.	(76, 85)
Opportunistic taxa (*Prevotella*, *Porphyromonas*, *Dialister*, *Veillonella*, *Escherichia-Shigella*)	Dysbiosis-associated	↑ pre-CRT and in patients with gastrointestinal side effects, and high toxicity scores.	(14, 21, 38, 39, 60, 78, 84)

(This table summarizes the microbial shifts in vaginal and gut microbiomes in locally advanced cervical cancer and chemoradiotherapy. CST = Community State Type, ↑ = increased, ↓ = decreased.).

### 3.5 Microbial influences on immunotherapy outcomes in cervical cancer

Given the increasing incorporation of immunotherapy into high-risk locally advanced cervical cancer treatment, we also reviewed the limited but emerging evidence on the influence of the microbiome on immunotherapy. Although cancer immunotherapy has shown clinical benefits, including in cervical cancer, treatment resistance and immune-related adverse events remain challenging and can lead to discontinuation. Optimizing therapeutic efficacy while minimizing systemic, endocrine, dermatologic, and gastrointestinal toxicities is therefore essential (11, 12). As current evidence is largely derived from other malignancies, with limited data specific to cervical cancer, and often in metastatic tumors or recurrences, the available studies are summarized and discussed alongside insights from other tumor types.

Cheng et al. investigated the association between the gut microbiome and clinical response six months after initiation of anti-programmed cell death protein 1 (PD-1) immunotherapy in patients with advanced cancers (n=72), including cervical cancer (n=3). The response group exhibited higher gut microbial diversity pre- and post-immunotherapy. Prior to treatment, responders showed significantly greater abundances of the phyla Euryarchaeota and Lentisphaerae. Interestingly, the non-response group had higher levels of Clostridiaceae (87), a family generally considered beneficial due to its butyrate-producing members and association with gut health. This underscores the importance of examining associations at the genus or species level. Exploratory analyses from the phase II PRIMMO trial further support the association between gut microbiota composition and immunotherapy outcomes in gynecologic cancers. In patients with recurrent or metastatic cervical (n=15) and endometrial cancer (n=20) treated with a pembrolizumab-based regimen, baseline microbiome composition differed between patients with favorable and poor clinical outcomes. Enrichment of the genus *Blautia* was observed in responders, whereas poor outcomes were associated with enrichment of Enterobacteriaceae, *Clostridium*, and Clostridiaceae, consistent with earlier observations from Cheng et al. linking *Clostridiaceae* to non-response and suggesting potentially recurring taxonomic patterns across cohorts (88). Regarding treatment-related adverse events (TRAEs), no significant differences in gut microbiome diversity or community structure were observed at baseline between patients with and without grade ≥ 3 toxicities. However, at day 99, patients experiencing grade ≥ 3 gastrointestinal TRAEs showed significantly decreased α-diversity. Specific taxa, including *Prevotella, Holdemanella*, and *Roseburia* were associated with toxicity, whereas *Barnesiella* was enriched in patients without severe toxicities (88).

A retrospective cohort study evaluated the impact of antibiotic exposure before or during immunotherapy in women with gynecologic cancers (n=101), including cervical cancer patients (n=22). Notably, patients with cervical cancer demonstrated the highest rate of prior antibiotic use compared with those with ovarian or endometrial cancer. In cervical cancer patients, antibiotic use prior to immunotherapy was significantly associated with lower response and progression-free survival rates compared with patients who had no antibiotic exposure or received antibiotics concurrently with immunotherapy (progressive disease – 58.3% vs. 10.0%; stable disease – 33.3% vs. 40.0%; partial response 8.3% vs. 30.0%; complete response 0.0% vs. 20.0%; p = 0.04) (89). The negative impact of antibiotic treatment prior to or during immunotherapy is consistent with a meta-analysis across multiple cancer types, including melanoma and non-small-cell lung cancer, which reported that patients receiving antibiotics had significantly reduced progression-free survival (HR = 1.53, 95% CI 1.22–1.93; p < 0.01). Although sensitivity analyses using adjusted hazard ratios were performed, residual confounding and reverse causality could not be fully excluded (90).

These findings suggest that while gut microbiota composition is associated with immunotherapy response, and increased microbial diversity is consistently linked to favorable outcomes, specific microbial interactions appear to be tumor-dependent. This complicates direct comparison between studies and highlights the need for further research into microbiome-immunotherapy interactions in cervical cancer.

### 3.6 Modulation of the microbial environment

As several bacterial species and their metabolites are linked to beneficial effects within the tumor microenvironment, microbiota-based interventions may help to restore microbial balance, optimize therapeutic responses, and inhibit the colonization of antimicrobial-resistant pathogens in the cervicovaginal tract (60, 67). Since oral probiotics are the most extensively studied in this context, this review will primarily focus on their role. According to the Food and Agriculture Organization of the United Nations and the World Health Organization (FAO/WHO), probiotics are defined as “live microorganisms which, when administered in adequate amounts, confer a health benefit on the host” (91).

Oral probiotics are believed to modulate multiple factors involved in tumorigenesis. They exert general anticancer effects by activating natural killer (NK) cells and promoting the maturation of dendritic cells (DCs) (92, 93). Lactic acid bacteria, in particular, produce anti-tumor metabolites, including SCFAs, lactic acid, and bacteriocins. These metabolites play a role in regulating cell differentiation, inhibiting cancer cell migration, and regulating the immune response (92). Other key characteristics of probiotics include acid and bile tolerance, the ability to adhere to mucosal and epithelial cells, antimicrobial activity, and antitumor effects such as inducing apoptosis, inhibiting metastasis, reducing inflammation, and enhancing immune function (57, 59). By restoring gut microbiome homeostasis, eliminating pathogenic bacteria, strengthening the intestinal barrier through the production of SCFAs, and modulating immune responses, probiotics could reduce adverse effects of radiotherapy, including radiation-induced diarrhea (RID) (94).

Oral supplementation with certain species, including members of *Bifidobacterium, Lactobacillus*, and *Lactococcus*, can promote the conversion of linoleic acid into conjugated linoleic acid (CLA). CLA has been associated with broad anticancer and anti-inflammatory effects by suppressing carcinogenic bacteria, modulating enzyme activity, and potentially downregulating COX-2 expression, which is associated with cell proliferation and inflammation (47). Additionally, an oral *Bifidobacterium* cocktail (including *B. breve* and *B. longum)* has been shown to enhance the anti-tumor immune response by modulating dendritic cell function and increasing the accumulation of CD8+ T cells in tumor-bearing mice (95). However, findings from murine studies cannot be directly translated to humans due to significant differences in innate and adaptive immunity (96).

Recent studies have explored the effects and mechanisms of various oral probiotics on the efficacy and toxicity of (chemo)radiotherapy. The beneficial effects of *L. rhamnosus* include the maintenance of intestinal microbiota homeostasis, protection of the mucosa, restoration of the intestinal barrier, modulation of immune responses and anti-inflammatory effects. It also has been shown to reduce the incidence of radiotherapy-induced diarrhea (97). *L. acidophilus* and *Bifidobacterium bifidum* reduced radiation-induced diarrhea. In addition, *Lactobacillus* species, including *L. acidophils and L. casei*, enhanced the anti-proliferative and pro-apoptotic effects of chemoradiotherapy by upregulating the immune response (59). Moreover, results of studies investigating oral probiotic consumption during radiotherapy were summarized by Ashique et al. The administration of *L. acidophilus, L. casei, Bifidobacterium animalis, and Bifidobacterium bifidum* improved stool consistency and decreased the severity of diarrhea, along with reducing the need for anti-diarrheal medication (59).

Oral supplementation with *Lactobacillus delbrueckii subsp. lactis* during RT in 54 gynecological cancer patients prevented a decrease in the abundance of *Lactobacillus*. Supplementation with *L. delbrueckii* significantly reversed the reduction of vaginal *Lactobacillus* in patients receiving radical radiotherapy, however, this effect was not significant in patients who received adjuvant radiotherapy. It is hypothesized that radical treatment involves higher radiation doses, which induces greater toxicity. With more severe toxicity and, consequently, a more disturbed vaginal microenvironment, the restorative effect of *L. delbrueckii* administration may be more pronounced (61). In a randomized controlled trial (RCT) of 63 cervical cancer patients, oral supplementation with live *L. acidophilus* and *Bifidobacterium bifidum* significantly reduced both the incidence of radiation-induced diarrhea and the need for anti-diarrheal medication (98). Similarly, an RCT involving 229 pelvic cancer patients found that oral administration of *L. acidophilus* and *Bifidobacterium longum* decreased the incidence of radiation-induced diarrhea at the end of RT (99). Linn et al. reported comparable results, with combined oral *L. acidophilus* LA-5 and *Bifidobacterium animalis* subsp. lactis BB-12 reducing RID incidence (53.8% vs. 82.1% in placebo) and loperamide use (100). However, not all clinical trials demonstrated significant benefits of the administration of probiotics. In an RCT involving 85 gynecologic cancer patients receiving pelvic RT with weekly cisplatin, oral *L. casei* strain DN-114–001 did not significantly reduce diarrhea incidence or the use of rescue medication, although it delayed the onset of looser stools. In contrast to the above-mentioned studies reporting a reduction in RID, no consistent trend was observed in this trial (66).

In an RCT (n=490), Delia et al. reported that patients receiving an oral probiotic preparation containing lactobacilli, bifidobacteria and *Streptococcus salivarius* ssp. *thermophilus* during radiotherapy for pelvic cancers experienced significantly lower rates of RID, colitis, and severe gastrointestinal toxicity (grade 3-4), as well as reduced daily bowel movements and a delayed need for anti-diarrheal medication compared to placebo patients (101). It should be noted that the reported effects in above-mentioned studies were limited to the treatment period or short-term follow-up (up to 4 weeks post-radiotherapy), with no long-term follow-up data available. An overview of the reported studies is provided in **Table 3**.

**Table 3:** Oral probiotics in cervical cancer therapy: effects on anti-tumor responses and gastrointestinal side effects.

Oral Probiotics	Effects	References
*Lactobacillus* (in general)	↑ production of anti-tumor metabolites (SCFAs, lactic acid, bacteriocins); enhances effects of CRT. ↓ gastro-intestinal side effects.	(59, 92, 101)
*Lactobacillus acidophilus*	↓ radiation-induced diarrhea. ↓ need for anti-diarrheal medication.	(59, 98–100)
*Lactobacillus casei*	↓/= gastro-intestinal side effects. ↓/= radiation-induced diarrhea.	(59, 66)
*Lactobacillus delbrueckii*	↑ vaginal *Lactobacillus* in patients receiving radical radiotherapy.	(61)
*Lactobacillus rhamnosus*	↑ intestinal microbiota homeostasis, protection and restoration of intestinal barrier, anti-inflammatory effects. ↓ radiation-induced side effects.	(97)
*Bifidobacterium* (in general)	↑ anti-tumor immune response. ↓ gastro-intestinal side effects.	(95, 101)
*Bifidobacterium animalis*	↓ radiation-induced diarrhea. ↓ need for anti-diarrheal medication.	(59, 100)
*Bifidobacterium bifidum*	↓ radiation-induced diarrhea. ↓ need for anti-diarrheal medication.	(59, 98)
*Bifidobacterium longum*	↓ radiation-induced diarrhea.	(99)
*Streptococcus salivarius ssp. Thermophilus* (in combination with lactobacilli and bifidobacteria)	↓ gastro-intestinal side effects including radiation-induced diarrhea. ↓ need for anti-diarrheal medication.	(101)

(This table summarizes the probiotics, and their effects, that are discussed in this review. ↑ = increased, ↓ = decreased, = = no significant effect.).

Vaginal toxicities occur in approximately 39% of cervical cancer patients undergoing definitive chemoradiotherapy (95% CI: 21-56%), yet no studies have specifically investigated the potential effects of probiotics on these vaginal toxicities. Such toxicities, including stenosis, bleeding, and mucositis, can significantly affect patients’ quality of life (102). In an RCT involving 77 patients with nasopharyngeal cancer, an oral probiotic formulation containing multiple *Lactobacillus* spp. was found to reduce the severity of oral mucositis compared to a placebo (103). This suggests a promising direction for exploring the use of probiotics in mitigating vaginal toxicities.

In addition to oral probiotic supplementation, direct modulation of the vaginal microbiome represents an emerging strategy. Vaginal probiotics may theoretically provide a more targeted approach to restoring a *Lactobacillus*-dominated vaginal environment by delivering beneficial strains directly to the vaginal niche. However, evidence regarding long-term colonization, safety, and clinical efficacy remains limited. In an RCT among high-risk HPV-positive women without cervical cancer (n=100), vaginal administration of *Lactobacillus crispatus* chen-01 was associated with a reduction in HPV viral load and improved HPV clearance (104). Nevertheless, clinical evidence supporting the use of vaginal probiotics in cervical cancer patients, particularly during or after CRT, is currently lacking. Similarly, vaginal microbiota transplantation (VMT) has been proposed as an emerging strategy to restore a healthy vaginal microbial community. Studies suggest potential efficacy in the treatment of bacterial vaginosis. However, translation to cervical cancer remains unexplored. Important challenges include donor screening, safety assessment, and the lack of standardized protocols, which currently limit clinical implementation (105). Further research is required to determine whether vaginal microbiome-directed interventions can reduce treatment-related toxicity or improve therapeutic outcomes in cervical cancer.

Moreover, studies have investigated the effect of prebiotics on the gut microbiome and host health. Prebiotics, including dietary fibre, are non-digestible food components that are selectively utilized by beneficial microorganisms, thereby promoting the host health trough modulation of microbial composition and metabolic activity. They promote the growth of bacteria supporting intestinal mucosal barrier integrity, thereby contributing to the activation of metabolic pathways involved in immune regulation (106). Inulin and other dietary fibres promote the growth of beneficial bacteria, including *Lactobacillus* and *Bifidobacterium*, which can inhibit pathogenic species and reduce inflammation (107). However, an RCT (n=100) conducted by Sasidharan et al. evaluating oral resistant starch supplementation in cervical cancer patients found no significant differences in the severity of acute radiation proctitis or in stool SCFA concentrations (108). In contrast, a dietary intervention trial in 134 patients with LACC reported increased fibre intake in the intervention group, associated with a reduced risk of mild constipation and a non-significant trend toward less severe diarrhea (109).

Besides pre- and probiotics, postbiotics are emerging as potential adjuvant therapies in cancer. Postbiotics are defined as preparations of inanimate microorganisms and/or their components (e.g. SCFAs) that confer a health benefit to the host. They can be derived from foods or administered as supplements. Preclinical studies suggest that postbiotics may enhance intestinal barrier integrity and modulate host immunity (110).

Fecal microbiota transplantation (FMT) has also emerged as a potential adjunctive therapeutic strategy in cancers. Although FDA-approved for the treatment of recurrent *Clostridioides difficile* infection, large-scale RCTs in cancer patients are still lacking. FMT is hypothesized to restore microbial diversity, potentially improving gastrointestinal toxicity and restoring SCFA-producing taxa. However, current evidence remains limited and highly context- and host-dependent, particularly in cervical cancer (111, 112).

Beyond conventional microbiome modulation, bacteria are increasingly being investigated as novel therapeutic agents in oncology because of their ability to accumulate within tumors, modulate immune responses, and serve as vehicles for targeted drug delivery. However, these approaches remain largely preclinical, and challenges related to safety, delivery efficiency, and tumor heterogeneity continue to limit clinical translation (113).

Characterizing the tumor, gut and/or vaginal microbiome prior to treatment could open possibilities for personalized microbial interventions. With this approach, pathogenic bacteria that impair anti-tumor effects of therapy could be reduced, while introducing beneficial bacteria to optimize treatment response and side-effects (75). Taken together, current evidence suggests that the administration of pre- and probiotics may help restore and maintain microbial homeostasis during CRT, thereby mitigating gastrointestinal toxicity and potentially enhancing treatment efficacy in cervical cancer.

## 4 Discussion

Cervical cancer remains one of the most common malignancies among women worldwide. Despite advances in treatment, both short- and long-term toxicities continue to significantly impact quality of life. This narrative review summarizes current evidence on the role of the gut and vaginal microbiomes in locally advanced cervical cancer, with a focus on their potential clinical and therapeutic relevance.

Emerging evidence suggests that microbial composition may be associated with both cervical carcinogenesis and the efficacy and toxicity of cervical cancer therapy (14–18). However, most available evidence remains associative, and whether microbial alterations represent drivers of treatment response and treatment-related toxicity or are secondary consequences of therapy-induced tissue damage remains largely unsolved. This distinction is particularly relevant because CRT itself induces substantial changes in microbiome composition, making it difficult to determine whether specific microbial communities are predictive biomarkers, causal contributors, or markers of treatment-induced alterations.

The biological interpretation of microbiome alterations is further complicated by differences between microbial compartments. While crosstalk between the gut and vaginal microbiomes likely exists, their distinct characteristics should be considered. A healthy VMB is typically characterized by low microbial diversity, while high gut microbial diversity is generally considered beneficial. In particular, microbial communities characterized by a higher relative abundance of symbiotic bacteria, such as *Lactobacillus* and *Bifidobacterium* spp., have been associated with improved epithelial barrier integrity and the secretion of antimicrobial substances (21). Conversely, dysbiosis has been linked to chronic inflammation, which is associated with tumorigenesis (23). However, these associations should be interpreted cautiously, as the effects of individual bacterial taxa are highly context-dependent. The same bacterial species may exert different effects depending on strain-specific characteristics, local environmental conditions, and metabolic output. Therefore, associations between specific taxa and clinical outcomes should not automatically be interpreted as causal mechanisms.

A functional, metabolite-centered perspective may provide additional insight beyond taxonomic profiling alone. Microbial metabolites, including SCFAs, bile acids, and tryptophan-derived compounds, as well as bacterial components and interspecies interactions, can influence immune regulation, epithelial barrier function, and the tumor microenvironment. However, the effects of these metabolites are highly dependent on their local concentration and microbial environment. Therefore, understanding microbiome-mediated effects requires integration of microbial composition with functional characteristics rather than relying solely on the presence or absence of individual taxa.

Chemoradiotherapy induces measurable alterations in both the gut and vaginal microbiomes, often characterized by reduced microbial stability, loss of beneficial taxa, and enrichment of opportunistic pathogens (23, 61). Several studies suggest that the baseline microbiome may be associated with treatment outcomes, with greater gut microbial diversity and *Lactobacillus*-dominant vaginal communities being linked to more favorable clinical responses and increased immune infiltration (15, 22, 36). Nevertheless, these findings should be interpreted with caution, as microbial composition is influenced by multiple host and treatment-related factors, including antibiotic exposure and diet. Consequently, it remains unclear whether modifying the microbiome alone would be sufficient to improve treatment outcomes.

Given the microbiome-host interactions, we hypothesize that the microbiome may represent a modifiable factor in cervical cancer treatment to optimize therapeutic responses and mitigate side effects. Microbiome-targeted interventions, such as probiotics, prebiotics, postbiotics, or microbial-derived metabolites, could potentially serve as treatment strategies to restore or maintain microbial homeostasis during therapy. However, it remains unclear whether such interventions should follow a “one-size-fits-all” approach or be tailored to the individual baseline microbiome.

Probiotics are among the most extensively studied approaches, particularly for reducing gastrointestinal toxicity associated with pelvic radiotherapy. It has been suggested that oral probiotics may reduce radiation-induced diarrhea and improve tolerance during treatment. However, important limitations remain. Most probiotic studies have focused on gastrointestinal outcomes rather than vaginal toxicity, urinary toxicity, or oncological outcomes, and evidence demonstrating improved tumor response remains limited. Furthermore, available studies are heterogeneous with respect to probiotic strains, formulations, doses, and treatment schedules, making direct comparisons difficult.

Although oral probiotics have been proposed to influence the vaginal microbiome through a gut-vaginal axis, direct evidence demonstrating consistent vaginal colonization after oral administration remains limited. Nevertheless, oral administration currently represents the most feasible and clinically translatable approach due to its safety and ease of use. Emerging strategies such as vaginal probiotics and vaginal microbiota transplantation may provide another target for modulation of the vaginal ecosystem by directly influencing the local microbial environment. However, evidence for these approaches in cervical cancer patients undergoing CRT is lacking, and their safety, feasibility, and long-term effects require further investigation. Alternative strategies, including prebiotics, defined microbial metabolites, and postbiotic components, may offer additional opportunities for microbiome modulation and immune regulation. However, their clinical relevance remains uncertain, and further evaluation is required to understand how these approaches influence microbial interactions and host responses.

Future research should therefore focus on investigating changes in both the gut and vaginal microbiomes before, during, and after chemoradiotherapy, with and without oral or vaginal microbial interventions. These microbiome shifts should be correlated with patient-reported side effects, quality of life, response rates, and immunological parameters to gain a deeper understanding of the microbiome’s impact on cervical cancer treatment, and to determine to what extent adverse effects are driven by CRT itself, an unfavorable baseline microbiome or metabolic profile, or an interplay between these factors.

### Limitations

Several limitations of the available evidence should be acknowledged. First, the included studies are heterogeneous and generally limited by relatively small sample sizes. Moreover, most studies are observational in nature, precluding conclusions regarding causality. In addition, substantial methodological heterogeneity exists with respect to sample collection, sequencing protocols, bioinformatic pipelines, and definitions of microbial dysbiosis. These methodological differences should be considered when comparing studies and may partly explain the inconsistent findings reported in the literature. Finally, potential confounding factors, including antibiotic use, diet, and other host-related variables, are not consistently accounted for, despite their well-established influence on microbiome composition.

## 5 Conclusions

In conclusion, accumulating evidence suggests that the gut and vaginal microbiomes are associated with the development, progression, and therapeutic outcomes of cervical cancer. Microbial dysbiosis has been linked to chronic inflammation and impaired treatment responses, while a beneficial microbiome, dominated by symbiotic bacteria, seems associated with improved therapeutic efficacy and reduced side effects. Microbiome-based interventions, including probiotics, prebiotics, postbiotics, and/or microbial-derived metabolites, represent promising strategies to modulate host-microbiome interactions during chemoradiotherapy. Nonetheless, current evidence in cervical cancer remains largely observational, and prospective interventional studies are lacking. Therefore, microbiome-based interventions cannot yet be recommended as part of routine clinical care. Before implementation, standardized methodologies, validation of predictive microbial biomarkers, and randomized clinical trials are required to establish causality, identify patients most likely to benefit, and determine the optimal intervention strategies. For clinicians, the current evidence highlights the microbiome as a promising but not yet clinically actionable target. Future longitudinal studies incorporating both the gut and vaginal microbiomes, microbial and systemic metabolites, immune infiltrates, systemic inflammation, patient-reported outcomes, and treatment results are essential for assessing the potential of microbial intervention strategies in personalized cervical cancer care.
