# Supplementary material for: Microbiome-host interactions in locally advanced cervical cancer: the impact on chemoradiotherapy treatment outcomes and toxicity
Source: Front Immunol. 2026 Aug 25;17:1876914. doi: 10.3389/fimmu.2026.1876914 (PMC13551732; doi:10.3389/fimmu.2026.1876914)
Supplement: Supplementary file 1 [file SupplementaryFile1.docx]

Supplementary Material

# 1 Literature Search

**Final search PubMed**

| Concept | Search | Hits |
| --- | --- | --- |
| Cervical Cancer (Condition) | ("LACC"[Title/Abstract] OR "uterine cervical neoplasms"[MeSH Terms] OR "genital neoplasms, female"[MeSH Terms:noexp] OR (("cervi*"[Title/Abstract] OR "female genital"[Title/Abstract]) AND ("cancer*"[Title/Abstract] OR "tumo*"[Title/Abstract] OR "neoplas*"[Title/Abstract] OR "carcinoma*"[Title/Abstract] OR "malignan*"[Title/Abstract]))) | 188,790 |
| Microbiome (Other terms) | ("microbi*"[Title/Abstract] OR "VMB"[Title/Abstract] OR "dysbiosis"[Title/Abstract] OR "dysbiosis"[MeSH Terms] OR "disbiosis"[Title/Abstract] OR "bacteria*"[Title/Abstract] OR "dysbacteriosis"[Title/Abstract] OR "microflora"[Title/Abstract] OR "gastrointestinal microbiome"[MeSH Terms] OR "microbiology"[MeSH Subheading] OR "microbiota"[MeSH Terms]) | 2,326,854 |
| Chemoradiotherapy (Intervention) | ("chemoradi*"[Title/Abstract] OR "chemoradiotherapy"[MeSH Terms] OR "chemotherapy, adjuvant"[MeSH Terms] OR "adjuvant"[Title/Abstract] OR "neoadjuvant therapy"[MeSH Terms] OR "neoadjuvant"[Title/Abstract] OR "radiation"[Title/Abstract] OR "CRT"[Title/Abstract] OR "radiotherap*"[Title/Abstract] OR "irradiation"[Title/Abstract] OR "radiotherapy"[MeSH Subheading] OR "anticancer"[Title/Abstract] OR "anti-cancer"[Title/Abstract] OR "antitumo*"[Title/Abstract] OR "anti tumo*"[Title/Abstract] OR "antineoplastic"[Title/Abstract] OR "anti-neoplastic"[Title/Abstract]) | 1,518,977 |
| Combined | ("LACC"[Title/Abstract] OR "uterine cervical neoplasms"[MeSH Terms] OR "genital neoplasms, female"[MeSH Terms:noexp] OR (("cervi*"[Title/Abstract] OR "female genital"[Title/Abstract]) AND ("cancer*"[Title/Abstract] OR "tumo*"[Title/Abstract] OR "neoplas*"[Title/Abstract] OR "carcinoma*"[Title/Abstract] OR "malignan*"[Title/Abstract])))  AND  ("microbi*"[Title/Abstract] OR "VMB"[Title/Abstract] OR "dysbiosis"[Title/Abstract] OR "dysbiosis"[MeSH Terms] OR "disbiosis"[Title/Abstract] OR "bacteria*"[Title/Abstract] OR "dysbacteriosis"[Title/Abstract] OR "microflora"[Title/Abstract] OR "gastrointestinal microbiome"[MeSH Terms] OR "microbiology"[MeSH Subheading] OR "microbiota"[MeSH Terms])  AND  ("chemoradi*"[Title/Abstract] OR "chemoradiotherapy"[MeSH Terms] OR "chemotherapy, adjuvant"[MeSH Terms] OR "adjuvant"[Title/Abstract] OR "neoadjuvant therapy"[MeSH Terms] OR "neoadjuvant"[Title/Abstract] OR "radiation"[Title/Abstract] OR "CRT"[Title/Abstract] OR "radiotherap*"[Title/Abstract] OR "irradiation"[Title/Abstract] OR "radiotherapy"[MeSH Subheading] OR "anticancer"[Title/Abstract] OR "anti-cancer"[Title/Abstract] OR "antitumo*"[Title/Abstract] OR "anti tumo*"[Title/Abstract] OR "antineoplastic"[Title/Abstract] OR "anti-neoplastic"[Title/Abstract]) | 1,306 |

**Final search Embase (via OVID)**

| Concept | Search | Hits |
| --- | --- | --- |
| Cervical Cancer (Condition) | (exp uterine cervix tumor/ or female genital tract tumor/ or ("LACC" or (("cervi*" or "female genital") and ("cancer*" or "tumo*" or "neoplas*" or "carcinoma*" or "malignan*"))).ti,ab,kf.) | 259,134 |
| Microbiome (Other terms) | (exp dysbiosis/ or exp intestine flora/ or exp microbiology/ or exp microflora/ or ("microbi*" or "VMB" or "dysbiosis" or "disbiosis" or "bacteria*" or "dysbacteriosis" or "microflora").ti,ab,kf.) | 2,074,949 |
| Chemoradiotherapy (Intervention) | (exp chemoradiotherapy/ or exp adjuvant therapy/ or exp neoadjuvant therapy/ or rt.fs. or ("chemoradi*" or "adjuvant" or "neoadjuvant" or "radiation" or "CRT" or "radiotherap*" or "irradiation" or "anticancer" or "anti-cancer" or "antitumo*" or "anti-tumo*" or "antineoplastic" or "anti-neoplastic").ti,ab,kf.) | 2,240,999 |
| Combined | ((exp uterine cervix tumor/ or female genital tract tumor/ or ("LACC" or (("cervi*" or "female genital") and ("cancer*" or "tumo*" or "neoplas*" or "carcinoma*" or "malignan*"))).ti,ab,kf.)  AND  (exp dysbiosis/ or exp intestine flora/ or exp microbiology/ or exp microflora/ or ("microbi*" or "VMB" or "dysbiosis" or "disbiosis" or "bacteria*" or "dysbacteriosis" or "microflora" or "gastrointestinal microbiome").ti,ab,kf.)  AND  (exp chemoradiotherapy/ or exp adjuvant therapy/ or exp neoadjuvant therapy/ or rt.fs. or ("chemoradi*" or "adjuvant" or "neoadjuvant" or "radiation" or "CRT" or "radiotherap*" or "irradiation" or "anticancer" or "anti-cancer" or "antitumo*" or "anti-tumo*" or "antineoplastic" or "anti-neoplastic").ti,ab,kf.))  NOT "conference abstract".pt. | 920 |

**Final search Web of Science**

| Concept | Search | Hits |
| --- | --- | --- |
| Cervical Cancer (Condition) | TS=("LACC" or (("cervi*" or "female genital") and ("cancer*" or "tumo*" or "neoplas*" or "carcinoma*" or "malignan*"))) | 185,715 |
| Microbiome (Other terms) | TS=("microbi*" or "VMB" or "dysbiosis" or "disbiosis" or "bacteria*" or "dysbacteriosis" or "microflora" or "intestinal flora") | 2,085,177 |
| Chemoradiotherapy (Intervention) | TS=(“chemoradi*” OR “adjuvant” OR “neoadjuvant” OR “radiation” OR “CRT” OR “radiotherap*” OR “irradiation” OR “anticancer” OR “anti-cancer” OR “antitumor*” OR “anti-tumo*” OR “antineoplastic” OR “anti neoplastic”) | 2,827,760 |
| Combined | (TS=("LACC" or (("cervi*" or "female genital") and ("cancer*" or "tumo*" or "neoplas*" or "carcinoma*" or "malignan*"))))  AND  (TS=("microbi*" or "VMB" or "dysbiosis" or "disbiosis" or "bacteria*" or "dysbacteriosis" or "microflora" or "gastrointestinal microbiome" or "intestinal flora"))  AND  (TS=(“chemoradi*” OR “adjuvant” OR “neoadjuvant” OR “radiation” OR “CRT” OR “radiotherap*” OR “irradiation” OR “anticancer” OR “anti-cancer” OR “antitumor*” OR “anti-tumo*” OR “antineoplastic” OR “anti neoplastic”)) | 718 |

**Final search Clinicaltrials.gov**

| Concept | Search | Hits |
| --- | --- | --- |
| Cervical Cancer (Condition) | (LACC OR ((cervix OR cervical OR female genital) and (cancer OR cancers OR tumor OR tumors OR neoplasm OR neoplasms OR carcinoma OR carcinomas OR malignancy or malignancies)) | 18,989 |
| Microbiome (Other terms) | (microbiome OR microbiota OR VMB OR dysbiosis OR disbiosis OR bacteria OR dysbacteriosis OR microflora OR intestinal flora) | 13,916 |
| Chemoradiotherapy (Intervention) | (chemoradiotherapy OR adjuvant OR neoadjuvant OR radiation OR CRT OR radiotherapy OR irradiation OR anticancer OR anti-cancer OR antitumor OR anti-tumor OR antineoplastic OR anti neoplastic) | 70,495 |
| Combined | (LACC OR ((cervix OR cervical OR female genital) and (cancer OR cancers OR tumor OR tumors OR neoplasm OR neoplasms OR carcinoma OR carcinomas OR malignancy or malignancies)))  AND  (microbiome OR microbiota OR VMB OR dysbiosis OR disbiosis OR bacteria OR dysbacteriosis OR microflora OR intestinal flora)  AND  (chemoradiotherapy OR adjuvant OR neoadjuvant OR radiation OR CRT OR radiotherapy OR irradiation OR anticancer OR anti-cancer OR antitumor OR anti-tumor OR antineoplastic OR anti neoplastic) | 130 |
